# Be a Mom, a Web-Based Intervention to Prevent Postpartum Depression: The Enhancement of Self-Regulatory Skills and Its Association With Postpartum Depressive Symptoms

**DOI:** 10.3389/fpsyg.2019.00265

**Published:** 2019-02-18

**Authors:** Ana Fonseca, Fabiana Monteiro, Stephanie Alves, Ricardo Gorayeb, Maria Cristina Canavarro

**Affiliations:** ^1^Center for Research in Neuropsychology and Cognitive-Behavioral Intervention, University of Coimbra, Coimbra, Portugal; ^2^Faculdade de Medicina de Ribeirão Preto, University of São Paulo, São Paulo, Brazil

**Keywords:** Be a Mom, emotion regulation abilities, pilot randomized trial, postpartum depression, prevention, psychological flexibility, self-compassion, web-based interventions

## Abstract

**Aim:** Be a Mom is a self-guided, web-based intervention to prevent persistent postpartum depression symptoms [PPD], targeting both at-risk postpartum women and/or women presenting early-onset postpartum depressive symptoms (selective/indicated preventive intervention). Be a Mom is grounded on the principles of Cognitive-Behavior Therapy and incorporates the recent contributions of acceptance and compassion-based approaches (third-wave approaches) applied to the perinatal context. This study aimed to explore the processes underlying therapeutic change in the Be a Mom intervention, by: (1) exploring whether participation in the Be a Mom promotes the enhancement of self-regulatory skills (emotion regulation abilities, psychological flexibility and self-compassion) in comparison with women who did not participate in the program; and (2) exploring whether changes in self-regulatory skills are associated with changes in depressive symptoms, among women who participated in the Be a Mom program.

**Methods:** A pilot randomized, two-arm controlled trial was conducted. Eligible women (presenting PPD risk-factors and/or early-onset PPD symptoms) were enrolled in the study and were randomly assigned to the intervention group (Be a Mom, *n* = 98) or to the waiting-list control group (*n* = 96). Participants in both groups completed baseline (T1) and post-intervention assessments (T2), including measures of depressive symptoms, emotion regulation abilities, psychological flexibility and self-compassion.

**Results:** From baseline to post-intervention assessment, women in the intervention group showed a significantly greater decrease in the levels of emotion regulation difficulties (*p* < 0.001) and a significant greater increase in the levels of self-compassion (*p* < 0.001) compared to the control group. No significant differences were found concerning psychological flexibility. Moreover, a greater decrease in difficulties in emotion regulation and greater increase in self-compassion levels were significantly associated with a greater decrease in depressive symptoms, among women in the intervention group.

**Discussion:** Be a Mom promotes the enhancement of women’s emotion regulation abilities and self-compassion, and this seems to exert a protective effect in the presence of PPD risk factors (or early-onset symptoms) because it led to a reduction of depressive symptoms. By providing some insights into the processes that underlie treatment response to Be a Mom, this study highlights the important role of the targeted third-wave processes applied to the perinatal context.

## Introduction

Postpartum depression (PPD) represents a significant public health problem. PPD is the most prevalent condition after childbirth and affects 13–20% of new mothers (Pearlstein et al., 2009; Gelaye et al., 2016), with estimates being higher in low- and middle-income countries (Fisher et al., 2012). Existing research has clearly identified a set of factors that put women at increased risk for developing PPD, including prior history of anxiety/depression, prenatal anxiety and depression, occurrence of stress-inducing events (e.g., death, divorce or job loss), lack of social support and poor quality of marital relationship (e.g., Beck, 2002; Robertson et al., 2004; Enatescu et al., 2017); this well-defined set of risk factors resulted in the development of risk inventories (e.g., Beck et al., 2006) that allow the timely identification of women at increased risk of PPD. Women who meet criteria for PPD often display comorbid anxiety symptoms (Falah-Hassani et al., 2016) and are at an increased risk for prolonged depression (Netsi et al., 2018). If untreated, the effects of PPD may be far-reaching and long-lasting, not only to the mother’s health (Woolhouse et al., 2014), but also to the mother-child interaction (Tronick and Reck, 2009) and to the child’s development (Kingston et al., 2012; Stein et al., 2014). Given the significant number of maternal-infant dyads affected by PPD (Drury et al., 2016), there have been increased efforts to implement effective prevention and treatment approaches to target this clinical condition.

Existing preventive interventions for PPD have been found to be effective (Sockol, 2015), although the effects were modest when compared to PPD treatments. Most existing face-to-face preventive interventions focus on minimizing PPD risk factors (e.g., lack of social support) without grounding in psychological therapy models, despite evidence of their increased effectiveness (Clatworthy, 2012; Werner et al., 2015). Therefore, there is a need to develop preventive interventions for PPD grounded in well-established psychotherapeutic models with proven effectiveness in preventing and treating PPD, such as Cognitive-Behavior Therapy (CBT) (Sockol, 2015). Moreover, due to stigma and practical barriers, such as access constraints to healthcare services, women’s compliance with face-to-face preventive and treatment interventions for PPD is low (McGarry et al., 2009; Fonseca et al., 2015). Web-based interventions seem to be a feasible option for PPD prevention and treatment due to their characteristics (e.g., flexibility of access, privacy), and there is preliminary evidence of its efficacy (Lee et al., 2016), although further research is needed.

### Preventing PPD: Which Core Psychological Processes Should Be Targeted?

Despite the extensive body of research on contextual (e.g., socioeconomic status, marital status, lack of social support) and clinical (e.g., prior history of depression) risk factors for PPD (Robertson et al., 2004; O’Hara and McCabe, 2013), there are some recent studies (e.g., Haga et al., 2012; Felder et al., 2016; Fonseca et al., 2018b) linking the absence of self-regulatory skills, such as emotion regulation abilities, psychological flexibility, and self-compassion, to the development and maintenance of PPD. Emotion regulation abilities may be defined as the individual’s ability to be aware of and to understand their emotional states and to use flexible and situationally appropriate strategies to address emotions and engage in goal-directed behaviors (Gratz and Roemer, 2004). Individual differences in the regulation of emotions have been linked to a heightened risk for the onset of major depressive episodes (Joorman and Siemer, 2014), particularly individual differences in the ability to repair and regulate negative emotions, which may result in longer episodes of sadness and depressed mood (Nolen-Hoeksema et al., 2008). Specifically, the use of maladaptive emotion regulation strategies (e.g., rumination and suppression) has consistently been linked with the onset and maintenance of depressive symptoms (Nolen-Hoeksema et al., 2008; Joormann and Gotlib, 2010; Joorman and Vanderlin, 2014). In a longitudinal study that aimed to identify the predictors of PPD, Haga et al. (2012) found that the habitual use of maladaptive cognitive emotion regulation strategies (e.g., self-blame and rumination) was associated with postpartum depressive symptoms over time. Similar associations between maladaptive strategies of emotion regulation (e.g., suppression of emotional states) and maternal psychopathological symptoms were found in another recent study (Edwards et al., 2017). Moreover, women who presented clinically relevant postpartum depressive symptoms were found to present more emotion regulation difficulties than non-depressed women (Marques et al., 2018).

Psychological flexibility may be understood as the individual’s ability to accept aversive emotional experiences in the moment while maintaining engagement in value-based behaviors and choices (Hayes et al., 2006). Specifically, lower psychological flexibility has been found to be associated with higher depressive symptoms in the postpartum period (Zhu et al., 2015; Fonseca et al., 2018b). Evans et al. (2012) found that higher psychological flexibility is associated with higher maternal attachment, higher maternal responsiveness and lower postpartum depressive symptoms.

Finally, the role of self-compassion, understood as the individual’s ability to have a kind and caring attitude toward oneself in the face of personal inadequacies or suffering while acknowledging that all individuals share a common human condition (Neff, 2009, 2012), has also been highlighted. Lower levels of self-compassion were found to be associated with higher levels of postpartum depressive symptoms (Felder et al., 2016; Fonseca and Canavarro, 2018), and Cohen (2010) found that greater self-compassion during pregnancy can exert a protective effect on the development of postpartum depressive symptoms.

It seems that during the postpartum period, women need to establish a new identity as mothers while striving to regain a sense of normalcy within the context of rapid changes in their roles, responsibilities and self-image (Kanotra et al., 2007). This can challenge their emotional adjustment and translate into more negative thoughts and emotions. Women who present more difficulties in regulating their emotional states, lower psychological flexibility and lower self-compassion seem to be at higher-risk of developing persistent postpartum depressive symptoms. The promotion of women’s self-regulatory skills may help them to relate to themselves in a way that assists them with the changes and challenges they are experiencing (Woekel, 2011), allowing them to be aware of and non-judgmentally accept their negative private parenting-related experiences (e.g., self-doubts, fear, and sadness), to have a more compassionate attitude toward their experiences and difficulties, and to manage to act in ways that promote good parenting practices and mother-child relationships (Woekel, 2011; Haga et al., 2012; Burke and Moore, 2015). These skills can globally translate into better postpartum adjustment.

### Be a Mom: A Web-Based CBT Intervention to Prevent PPD

Be a Mom is a short-term, self-guided, web-based selective/indicated preventive intervention that targets women at-risk for PPD or those who present early-onset PPD symptoms (i.e., present scores above the cutoff score in instruments assessing risk factors for PPD and/or in postpartum depression screening measures). It is grounded in CBT principles and incorporates the recent contributions of third-wave CBT approaches applied to the perinatal context namely self-compassion and acceptance and commitment therapies (Klausen, 2005; Cree, 2015). Be a Mom is a structured program that has a modular setup including five modules, with each module addressing one or two specific thematic contents (Changes and Emotional Reactions; Cognitions; Values and Social Support; Couple’s Relationship; PPD Alert Signs and Professional Help-seeking). The content of each module includes psychoeducational information about the specific thematic content and practical strategies to be implemented by the women during the following weeks. The information is presented in attractive formats (text, animation, and video) and through the incorporation of several content-related interactive exercises with personalized feedback on the user’s responses. The modules follow the structured and goal-oriented nature of CBT sessions: the module’s goals are presented, followed by the module’s thematic content (interchangeably with several interactive exercises), and a homework activity is presented at the end of each session to guarantee continued therapeutic practice. Although the Be a Mom program is completely self-guided in nature, asynchronous communication channels (e.g., reminders, email contact for program-related support) are available to enable communication.

Be a Mom targets the enhancement of core self-regulatory skills, such as emotion regulation, psychological flexibility and self-compassion, by helping women to: (a) be aware of, understand and non-judgmentally accept the diversity of their private experiences (emotions and thoughts) during the postpartum period; (b) use more psychologically flexible (e.g., acceptance, cognitive defusion) and self-compassionate ways to deal with such experiences; and (c) identify, create and clarify parenthood values while engaging in committed actions with such values (Fonseca et al., 2018c). Be a Mom also addresses perinatal-specific concerns (e.g., communication with the social network, the couple’s relationship), which have been found to be important dimensions for perinatal women (O’Mahen et al., 2012). The iterative formative evaluation process that informed the design and the intervention components of Be a Mom is detailed elsewhere (Fonseca et al., 2018c).

Cognitive-Behavior Therapy approaches targeting the development of self-regulatory skills such as self-compassion and psychological flexibility have been found to be effective for several mental health conditions (Powers et al., 2009; Leaviss and Uttley, 2015). However, to our knowledge, these approaches have seldom been investigated with regard to the prevention and treatment of PPD. We have conducted a pilot study aiming to gather evidence of the Be a Mom’s feasibility and acceptability, as well as preliminary evidence of Be a Mom’s efficacy, in terms of primary (depressive symptoms) and secondary (anxiety symptoms, maternal confidence, negative thoughts, marital satisfaction) outcomes. Preliminary evidence suggests that Be a Mom is effective in reducing early-onset postpartum depressive symptoms when compared with a waiting-list control group, and consequently in preventing the establishment of a clinical diagnosis of PPD (Fonseca et al., 2018a). Moreover, women in the Be a Mom group were also found to present a greater reduction in anxiety symptoms when compared to the waiting-list control group, although no differences were found in the remaining secondary outcomes (Fonseca et al., 2018a). However, this study focused in preliminary group comparisons to ascertain the program’s efficacy, without exploring the mechanisms of treatment response; thus, further research is needed to understand which processes are involved in the reduction of postpartum depressive symptoms among women who participated in the program and particularly whether the enhancement of self-regulatory skills may account for such reduction.

### The Present Study: Exploring the Processes of Change

Although most of the existing research on psychological interventions has focused on their effectiveness, there has been increased recognition of the need to understand not only *if* a psychotherapeutic intervention works but also *how* it works. Gaining a better understanding of the processes underlying therapeutic change is vital to optimize treatment outcomes and to allow the refinement of existing treatment procedures by establishing a clearer connection between the target of an intervention and its observed outcomes (Hayes et al., 2007; Kazdin, 2007; Lemmens et al., 2016). The purpose of this study is to explore the processes underlying therapeutic changes in the Be a Mom intervention, which has shown preliminary evidence of its efficacy in a pilot RCT reported elsewhere (Fonseca et al., 2018a). Although the pilot trial was designed to examine the acceptability, feasibility and efficacy of the Be a Mom program, a secondary analysis of the gathered data allows an exploratory inspection of the processes underlying therapeutic change, by examining changes in self-regulatory skills over time and its association with the primary outcome (depressive symptoms). Specifically, this study aims to: (1) explore whether the participation in the Be a Mom program promotes the enhancement of self-regulatory skills by examining changes in self-regulatory skills among women who participated and those who did not participate in the program; and (2) examine whether changes in each of the self-regulatory skills (emotion regulation abilities, psychological flexibility, and self-compassion) are associated with changes in the outcome (depressive symptoms), among women who participated in the Be a Mom program.

## Methods

### Study Design and Procedure

The trial is registered at clinicaltrials.gov (NCT03024645) and was approved by the Ethics Committees of Faculty of Psychology and Educational Sciences of University of Coimbra and of Centro Hospitalar e Universitário de Coimbra, EPE [CHUC]. This was a two-arm, open-label, pilot randomized controlled trial to assess the effectiveness of a web-based psychological intervention for preventing the establishment of a clinical diagnosis of PPD among at-risk women. Women were eligible to participate in the study if they were adult (≥18 years), in the early postpartum period (up to 3 months postpartum), and presented risk factors for PPD (i.e., a score equal to or above the cutoff score of 5.5 on the Postpartum Depression Predictors Inventory-Revised; Alves et al., 2018) and/or early-onset PPD symptoms (i.e., a score above the cutoff score of 9 in the Edinburgh Postpartum Depression Scale; Areias et al., 1996). Moreover, they needed to have access to a computer/tablet/smartphone and internet access at home and the ability to read and speak Portuguese and to be a Portuguese resident. The exclusion criteria were the presence of a serious medical condition (physical or psychiatric) in the mother or the infant (self-reported).

Women were recruited both in person (at the Maternity Daniel de Matos – CHUC, women were invited to participate in the study during their postpartum hospitalization and provided their contact information to be contacted by the researchers 4–6 weeks postpartum for assessment for eligibility criteria) and online (on social media websites, both through unpaid cross-posting and through paid boosting campaigns; women completed a web form where they provided their contact information to be contacted by the researchers 1–2 weeks later to be assessed for eligibility criteria). In both cases, the study goals and procedures were described, and the participants’ and researchers’ roles were clarified. All women gave their informed consent to participate in the study. Participants’ enrollment in the study occurred between June 2017 and October 2017.

Participants who met the eligibility criteria were randomly assigned (simple randomization procedure; allocation rate 1:1) to either the intervention group with access to the Be a Mom program or to the wait-list control group. Randomization was assured by a third researcher (different from the two responsible for enrollment and assignment of the participants to the study groups) who had no information about the participants (except for their code). The randomization sequence was concealed from the two researchers responsible for the participants’ enrollment and assignment to groups.

The study variables were assessed at baseline (Time 1 – T1) and post-treatment (Time 2 – T2). In the intervention group, women who completed Be a Mom received an automatic email inviting them to complete post-treatment assessments 2–3 days later. If women dropped out of the program, an automatic email was sent 8 weeks after registration. In the waiting-list control group, an email inviting participants to complete the assessment protocol was sent 8 weeks after T1. T1 and T2 assessment protocols were collected online through a survey platform (Limesurvey^®^) with secure access that prevented the same user from completing the survey more than once. The CONSORT 2010 guidelines and their extensions for pilot trials (Eldridge et al., 2016) and for ehealth (Eysenbach and Group, 2011) were considered.

### Intervention and Control Arms

The intervention arm had access to the Be a Mom program, which was described above. Women assigned to the intervention arm were invited by email to register on the Be a Mom platform (beamom.pt; access to the program is restricted to invitation). Access to the program was free of cost, and no compensation was given to participants. Only after registering in the Be a Mom’s platform did the women have access to the five modules, which had to be accessed in order. The participants were instructed that they should complete one module per week, although a slower place was allowed. They were also given the option of pausing the module and resuming the last page visited during subsequent access. Be a Mom includes only asynchronous communication channels (one reminder by email after 7 days without accessing Be a Mom; email contact for technical support). Participants in the waiting-list control arm were offered no intervention but were free to access other forms of care (as were all participants). At the end of the pilot trial, participants in the control group were offered the opportunity to access Be a Mom.

### Measures

#### Sociodemographic and Clinical Information

The women answered a self-report form including questions about sociodemographic (e.g., age, marital status, educational level, professional status, average monthly income, socioeconomic status and residence) and clinical data (e.g., psychopathological history, psychological/psychiatric treatment history). Infant’s data (e.g., age, gender, gestational weeks at birth) were also collected.

#### Postpartum Depression Risk

The Portuguese version of the Postpartum Depression Predictors Inventory-Revised (PDPI-R, postnatal version; Alves et al., 2018) is an inventory developed to assess PPD risk factors (e.g., low socioeconomic status, low self-esteem, prenatal depression/anxiety, lack of social support, and child care stress) and was used to identify women who are at higher risk for developing PPD. The questionnaire is composed of 39 items, answered on a dichotomous scale (yes vs. no, except for the first two items in which participants report their marital and economic status). The PDPI-R total score ranges from 0 to 39, with higher scores indicating increased risk for PPD. In the Portuguese validation studies, a score of 5.5 or higher is indicative of higher PPD risk (Alves et al., 2018).

#### Depressive Symptoms

The Edinburgh Postpartum Depression Scale [EPDS] (Cox et al., 1987; Areias et al., 1996) was used to assess depressive symptoms (e.g., sadness and tearfulness), in terms of their presence and severity. The women were asked to rate, using a 4-point scale, how frequently they felt different symptoms in the previous 7 days. Higher scores were indicative of higher depressive symptoms. According to the Portuguese validation studies, a score above 9 is indicative of clinically relevant depression symptoms. In the present study, Cronbach’s alpha values ranged from 0.74 (intervention group – T2) to 0.90 (control group – T1).

#### Emotion Regulation Abilities

To assess the women’s emotion regulation abilities, the short version of the Difficulties in Emotion Regulation Scale [DERS-SF] (Kaufman et al., 2015) was used. The DERS-SF is a self-report questionnaire to assess difficulties in using adaptive emotional regulation strategies and comprises 18 items (e.g., “When I’m upset, I feel guilty for feeling that way”) answered on a 5-point Likert scale (from 1 = Almost Never Applies to Me to 5 = Almost Always Applies to Me). The DERS-SF is organized into six dimensions (i.e., Non-acceptance of emotional responses, Lack of emotional awareness, Lack of emotional clarity, Difficulties engaging in goal-directed behaviors, Impulse control difficulties, and Limited access to emotion regulation strategies). It is also possible to compute a total score by summing all items, which was the approach used in this study. Higher scores were indicative of more difficulties in using adaptive emotional regulation strategies (i.e., less emotion regulation abilities). Cronbach’s alpha values ranged from 0.85 (intervention group – T2) to 0.91 (control group – T1).

#### Psychological Flexibility

To assess psychological flexibility, the Acceptance and Action Questionnaire-II [AAQ-II] (Bond et al., 2011; Pinto-Gouveia et al., 2012) was used. The AAQ-II comprises 7 items that measure the individual’s degree of psychological inflexibility (i.e., the degree of experiential avoidance of inner negative experiences, e.g., “I’m afraid of my feelings”) answered on a 7-point Likert scale (ranging from 1 = Never True to 7 = Always True). Higher scores are reflective of lower psychological flexibility (i.e., higher psychological inflexibility). In our sample, the Cronbach’s alpha values ranged from 0.90 (intervention group – T1 and T2) to 0.92 (control group – T1).

#### Self-Compassion

The short version of the Self-Compassion Scale [SCS-SF] (Castilho et al., 2015) was used to measure the women’s levels of self-compassion. The SCS-SF comprises 12 items (e.g., “When I’m going through a very hard time, I give myself the caring and tenderness I need”) answered on a 5-point response scale (ranging from 1 to 5). The 12 items measure six components (i.e., self-kindness, self-judgment, common humanity, isolation, mindfulness, and overidentification). It is also possible to compute a total self-compassion score. Higher scores are indicative of higher self-compassion. The Cronbach’s alpha values in our sample ranged from 0.84 (intervention group – T2) to 0.92 (control group – T1).

### Data Analyses

Preliminary analyses to characterize the sample, the study variables and the pattern of missingness were conducted with the *Statistical Package for the Social Sciences* (IBMS SPSS, version 22.0). The Latent Change Score (LCS) models were estimated by maximum likelihood (ML) using the Mplus program, version 7 (Muthén and Muthén, 1998-2017).

For sample characterization, descriptive statistics and comparison tests (*t*-tests and chi-square tests, respectively, for continuous and categorical variables) were used to compare the sociodemographic and clinical characteristics between the intervention and control groups. Pearson correlations were computed to examine the associations between sociodemographic and study variables.

Missing endpoints at posttest ranged from 48/194 (24.7%) on EPDS to 55/194 (28.4%) on DERS-SF (Little’s MCAR test *X*^2^_21_ = 28.57, *p* = 0.125). Missing data were handled using the Full Information Maximum Likelihood (FIML) estimation, which draws on all available data to estimate model parameters without imputing missing values (Enders, 2010).

For each variable (emotion regulation abilities, psychological flexibility and self-compassion), univariate LCS (McArdle, 2009) were computed to summarize longitudinal data and to examine changes over time. In contrast with comparison analyses (which examine differences between people in two or more groups), LCS models focus on examining changes within people over time, considering a within-subjects approach (McArdle, 2009). The LCS specification is a structural equation modeling approach to modeling data that can represent change over time with either manifest or latent measures of a time-dependent outcome (McArdle, 2001, 2009).

Change between T1 and T2 was modeled as a latent factor (not directly measured; defined as the part of the score of the variable at T2 that is not identical to the score of the variable at T1, i.e., the difference between scores at T1 and T2), which allows the estimation of: (a) the mean/intercept of the change between T1 and T2 (μ_Δ_, the average change over time, latent factor; a significant positive mean/intercept of the LCS factor suggests that, on average, an individual’s scores increased from T1 to T2, while a significant negative mean/intercept suggests that the individual’s scores decreased over time); (b) the variance/residual variance of the change between T1 and T2 (σ^2^_Δ_, the extent to which individuals differ in the change they manifest over time; a significant variance/residual variance in the LCS factor suggests heterogeneity across individuals regarding the averaged trajectory); (c) the covariance between the individual’s score at T1 and the latent change factor (σ_1Δ_); and (d) the mean scores at T1 (McArdle, 2009; Henk and Castro-Schilo, 2016). To better illustrate the meaning of such estimates, we can bear in mind the following example considering the variable *X*. Each individual has a different score in the variable *X* at T1 and at T2. For each individual, it is possible to compute a change score (difference between T1 and T2). Considering the group of individuals, it is possible to compute a mean of the change scores (μ_Δ_, the average change over time) and the deviation of each individual’s change score from the mean of change scores (σ^2^_Δ_), as well as the mean scores at T1. Finally, it is possible to examine the correlation between each individual’s scores at T1 and his/her change score over time.

A multigroup model approach was used to check for differences between the intervention and the control group in the four key parameters estimated. First, a fully constrained model was computed in which all the previously mentioned parameters were constrained to be equal across groups. Second, an unconstrained model was computed in which the key parameters were allowed to vary across groups. If the unconstrained model showed a better fit to data than the constrained the model, than the parameters estimated (e.g., mean/intercept of the change score) are significantly different across groups. To allow for model identification and comparison, one of the parameters (the covariance between the individual’s score at T1) remained fixed to be equal across groups.

**FIGURE 1:**
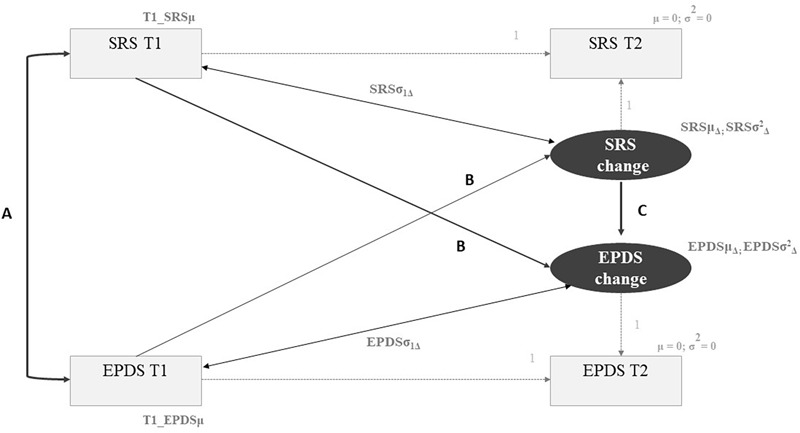
Two-wave Latent Change Score Model. Light gray parameters correspond to the estimations of the univariate Latent Change Score model. SRS, self-regulatory skill (emotion regulation abilities, psychological flexibility, self-compassion). EDPS, depressive symptoms. T1, baseline assessment. T2, post-intervention assessment. Parameter A, Correlation between individual’s scores at T1. Parameter B, Cross-lagged paths between T1 scores in one variable and the change score in the other variable. Parameter C, Change-to-change effect (effect of the change in one variable on the change of the other variable).

Finally, to examine whether changes in each of the self-regulatory skills were associated with changes in women’s depressive symptoms over time in the intervention group, three two-wave LCS models (2W-LCS; Henk and Castro-Schilo, 2016; Kievit et al., 2017; Valente and MacKinnon, 2017) were conducted. The graphical representation of the 2W-LCS model is depicted in Figure 1. In addition to the estimation of the four key parameters mentioned above (univariate LCS for each variable), this model allows the estimation of three additional parameters: (a) the correlation between an individual’s scores on the self-regulatory skill and depressive symptoms at T1; (b) the cross-lagged paths between T1 scores and the change score (i.e., the association between T1 scores of one variable and the change factor of the other variable); and (c) the change-to-change effect (i.e., the estimate of the effect of the change in one variable on the change of the other variable; a positive regression coefficient indicates that higher change scores in a variable are associated with higher change scores in the other variable, while a negative regression coefficient indicates that higher change scores in a variable are associated with lower change scores in the other variable).

We first tested a baseline model in which all three parameters were fixed to be zero. We gradually unconstrained the three parameters and examined changes in the model’s fit in comparison with the prior model.

The goodness-of-fit of the models was assessed by relying on different criteria: a non-significant chi-square statistic (*p* > 0.05), CFI above 0.95 and a Root Mean Square Residuals Standardized (SRMR) below 0.08. Comparison between competing models was made performed based on chi-square difference tests (significant Δ*X*^2^) and by comparing the goodness-of-fit indices of each model (Hu and Bentler, 1998, 1999; Kline, 2011). Significance was set at the level *p* < 0.05.

## Results

### Participants

**FIGURE 2:**
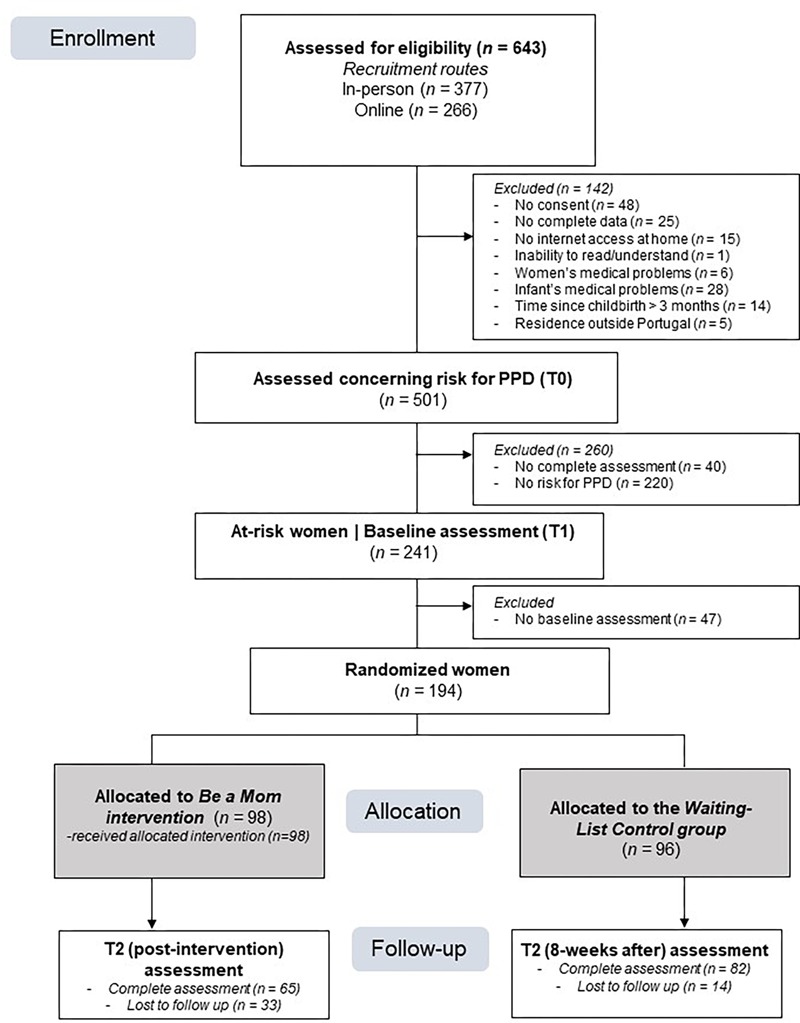
Flowchart of participants in the study.

The flow of participants through the study is presented in Figure 2. Of the 643 women enrolled in the study, 142 were excluded, and 501 were assessed with regard to the presence of PPD risk and/or early-onset PPD symptoms (T0). Of these, 48% of women (*n* = 241) presented risk for PPD or early-onset PPD symptoms and were given baseline assessments, with a participation rate of 80.5%.

One hundred and ninety-four at-risk women were randomized, with 98 allocated to the intervention group (Be a Mom) and 96 allocated to the waiting-list control group. The baseline sociodemographic and clinical characteristics of the participants are presented in Table 1. Comparison analyses showed that the control group had a higher proportion of single women and of women with an average income lower than 1000¬ (cf. Table 1). No additional differences were found. Associations between sociodemographic variables (marital status and income) and self-regulation skills were low and non-significant, except for the association between income and self-compassion (T1: *r* = -0.204, *p* = 0.004; T2: *r* = -0.214, *p* = 0.011). Income was introduced as a covariate in the models examining changes in self-compassion over time. Of the 98 women allocated to the intervention group, 41.8% (*n* = 41) completed the program.

**Table 1:** Participants’ sociodemographic and clinical characteristics at baseline.

	Intervention group (*n* = 98)	Control group (*n* = 96)	*t*/*X*^2^
**Women’s sociodemographic characteristics**			
Age (in years), *M* (*SD*)	32.22 (4.36)	32.94 (5.24)	1.03
Relationship, *n* (%)			
Married/living together	88 (89.8)	75 (78.1)	9.08^∗^
Single	4 (4.1)	16 (16.7)	
Divorced	2 (2.0)	3 (3.1)	
In a relationship (not living together)	4 (4.1)	2 (2.1)	
**Number of children, *n*** (%)			
Primiparous	62 (63.3)	71 (74.0)	4.07
Multiparous (prior children)	36 (36.7)	25 (26.0)	
**Educational level, *n* (%)**			
Basic education (9th grade)	6 (6.1)	5 (5.2)	0.76
Secondary education	22 (22.4)	25 (26.0)	
Higher education	42 (42.9)	43 (44.8)	
Postgraduate education (M.Sc.; Ph.D.)	28 (28.6)	23 (24.0)	
**Professional status, *n* (%)**			
Employed	82 (83.7)	76 (79.2)	0.81
Unemployed/Other	16 (16.3)	20 (20.8)	
**Monthly income, *n* (%)**			
Less than 500¬	3 (3.1)	5 (5.2)	57.78^∗∗^
500¬–1000¬	16 (16.3)	50 (52.1)	
1000¬–2000¬	51 (52.0)	17 (17.1)	
2000¬–3500¬	22 (22.4)	3 (3.1)	
More than 3500¬	6 (6.1)	21 (21.9)	
**Socioeconomic status, *n* (%)**			
Low	13 (13.3)	14 (14.6)	2.17
Medium/high	85 (86.7)	82 (85.4)	
**Residence, *n* (%)**			
Urban	74 (75.5)	70 (72.9)	0.17
Rural	24 (24.5)	26 (27.1)	
**Women’s clinical characteristics**			
Psychopathology history, *n* (%)			
Yes	53 (54.1)	44 (45.8)	1.32
No	45 (45.9)	52 (54.2)	
**Infant’s characteristics**			
Infant’s Gender, *n* (%)			
Male	58 (59.2)	54 (56.2)	1.14
Female	40 (40.8)	42 (43.7)	
Infant’s age (in months), *M* (*SD*)	2.00 (0.83)	1.99 (0.95)	-0.08

*^∗^p < 0.05, ^∗∗^p < 0.01.*

### Change in Self-Regulatory Skills Over Time: Comparisons Between Intervention and Control Groups

A univariate LCS model was tested for each self-regulatory skill (emotion regulation abilities, psychological flexibility, and self-compassion) to examine changes over time. A multigroup model approach was used to check for differences between the intervention and the control groups in the average of change over time and its variance. The full constrained model (in which the LCS estimates were constrained to be equal across groups) was compared with the unconstrained model (in which the LCS parameters were free to vary across groups). Descriptive statistics and LCS estimates for each group are presented in Table 2.

**Table 2:** Changes in self-regulatory skills over time in the intervention and control groups: Descriptives and Univariate Latent Change Scores estimates.

	Descriptives				Univariate latent Change Scores (LCS)			
	Intervention group		Control group		Intervention group		Control group	
	T1 *M (SD)*	T2 *M (SD)*	T1 *M (SD)*	T2 *M (SD)*	μ_Δ_	σ^2^_Δ_	μ_Δ_	σ^2^_Δ_
Diff. Emot. Reg.	40.80 (12.32)	36.95 (9.39)	36.99 (12.35)	34.74 (10.66)	-4.35^∗∗∗^	87.96^∗∗∗^	-2.05^∗∗^	51.29^∗∗∗^
Psych. Inflex.	21.05 (8.98)	18.52 (7.19)	19.63 (9.53)	18.37 (8.863)	-2.26^∗∗^	34.38^∗∗∗^	-1.34^∗^	36.32^∗∗∗^
Self-Compassion	37.65 (8.04)	40.91 (6.91)	41.02 (9.68)	43.05 (9.07)	3.46^∗∗∗^	45.99^∗∗∗^	1.75^∗∗^	30.33^∗∗∗^

*Diff. Emot. Reg., Difficulties in Emotion Regulation; Psych. Inflex., Psychological Inflexibility; T1, baseline assessment; T2, post-intervention assessment; μ_Δ_, mean/intercept of the latent change factor; σ^*2*^_Δ_, variance of the latent change factor. The LCS estimates presented correspond to the unconstrained model (where LCS parameters were free to vary across groups). Unstandardized estimates are presented. ^∗^p < 0.05, ^∗∗^p < 0.01, ^∗∗∗^p < 0.001*.

#### Emotion Regulation Abilities

Changes in emotion regulation difficulties from T1 to T2 were examined. The full constrained model presented a poor fit to the data (χ^2^_4_ = 22.10, *p* < 0.001, CFI = 0.85, SRMR = 0.08), while the unconstrained model presented an acceptable fit (χ^2^_1_ = 7.00, *p* = 0.008, CFI = 0.95, SRMR = 0.00). The comparison between models (Δχ^2^_3_ = 15.10, *p* < 0.001) suggested that there were significant differences across groups. The LCS estimates (see Table 2) showed that the levels of emotion regulation difficulties had a greater decrease from T1 to T2 in the intervention group than in the control group and that the changes were heterogeneous across individuals in both groups. The covariance between the levels of emotion regulation difficulties at T1 and the amount of change over time was also significant (σ_1Δ_ = -59.02, *SE* = 9.76, Z = -6.05, *p* < 0.001), suggesting that the higher the emotional regulation difficulties scores at T1, the greater the decrease in change scores.

#### Psychological Flexibility

Changes in psychological inflexibility from T1 to T2 were investigated. The full constrained model yielded a very good fit to the data (χ^2^_4_ = 1.94, *p* = 0.747, CFI = 1.00, SRMR = 0.04). The model fit of the unconstrained model was also very good (χ^2^_1_ = 0.43, *p* = 0.510, CFI = 1.00, SRMR = 0.03). The comparison between models (Δχ^2^_3_ = 1.51, *p* > 0.05) suggested that the more parsimonious model was the one in which the LCS estimates were constrained to be equal across groups. The LCS estimates suggested that the levels of psychological inflexibility decreased from T1 to T2 (μ_Δ_ = -1.78, *SE* = 0.48, *Z* = -3.67, *p* < 0.001) and that the changes were heterogeneous across individuals (σ^2^_Δ_ = 35.60, *SE* = 4.24, *Z* = 8.40, *p* < 0.001). The covariance between the levels of psychological inflexibility at T1 and the amount of change over time was significant (σ_1Δ_ = -28.13, *SE* = 5.01, *Z* = -5.61, *p* < 0.001), suggesting that the higher the levels of psychological inflexibility at T1, the greater the decrease in change scores.

#### Self-Compassion

With regard to changes in self-compassion levels from T1 to T2, income was first introduced in the constrained model as a covariate, but no significant associations with self-compassion levels at T1 (*p* = 0.07) or T2 (*p* = 0.867) were found. Therefore, it was removed from the model, to decrease the number of parameters to be estimated. The full constrained model presented an acceptable fit to the data (χ^2^_4_ = 14.14, *p* = 0.007, CFI = 0.92, SRMR = 0.16), while the unconstrained model presented a very good fit (χ^2^_1_ = 1.71, *p* = 0.191, CFI = 0.99, SRMR = 0.00). The comparison between models (Δχ^2^_3_ = 12.43, *p* < 0.001) suggested that there were significant differences across groups. The LCS estimates (see Table 2) showed that the intervention group had a greater increase in self-compassion levels from T1 to T2 than the control group. The covariance between the levels of self-compassion at T1 and the amount of change over time was significant (σ_1Δ_ = -26.82, *SE* = 4.87, *Z* = -5.51, *p* < 0.001), suggesting that lower scores of self-compassion at T1 were associated with a greater increase in change scores.

### Association Between Changes in Self-Regulatory Skills and Depressive Symptoms in the Intervention Group

Three two-wave LCS models were estimated to examine the effects of changes in each self-regulatory skill on changes in depressive symptoms. Table 3 presents the unstandardized parameter estimates from the two-wave LCS model for each of the estimated models.

**Table 3:** Change-to-change effects of self-regulatory skills on depressive symptoms: unstandardized parameter estimates from the Two-Wave Latent Change Score models.

	Model 1: Difficulties in emotion regulation and depressive symptoms		Model 2: Psychological inflexibility and depressive symptoms		Model 3: Self-compassion and depressive symptoms	
	*B*	*SE*	*B*	*SE*	*B*	*SE*
**Means/intercepts**						
μ_Δ_ SRS	-4.38^∗∗∗^	1.20	-2.39^∗∗^	0.718	3.53^∗∗∗^	0.84
μ_Δ_ EPDS	-1.75^∗∗∗^	0.46	-1.954^∗∗∗^	0.464	-1.77^∗∗∗^	0.48
**Variance/residual variance**						
σ^2^_Δ_ SRS	104.58^∗∗∗^	17.76	36.46^∗∗∗^	6.54	50.30^∗∗∗^	8.78
σ^2^_Δ_ EPDS	13.47^∗∗∗^	2.24	14.59^∗∗∗^	2.37	13.93^∗∗∗^	2.26
**Correlations**						
T1 SRS, T1 EPDS	18.86^∗∗∗^	4.74	16.18^∗∗∗^	3.61	-13.07^∗∗∗^	3.17
T1 SRS, ΔEPDS	-6.56^∗^	2.75	-3.60	1.90	3.79	1.96
**Predictive path**						
ΔSRS → ΔEPDS	0.115^∗∗^	0.038	0.10	0.07	-0.14^∗^	0.06

*SRS, Self-regulatory skill (in model 1: difficulties in emotion regulation, in model 2: psychological inflexibility, in model 3: self-compassion). EDPS, Depressive symptoms. μ_Δ_, average change over time. σ^*2*^_Δ_, variance/residual variance of change. T1, Time 1. ΔEPDS, Latent change EPDS score. ΔSRS, Latent change self-regulatory skill score. ^∗^p < 0.05, ^∗∗^p < 0.01, ^∗∗∗^p < 0.001*.

#### Emotion Regulation Abilities and Depressive Symptoms

A model examining the effect of changes in emotional regulation difficulties on changes in depressive symptoms over time was estimated. A baseline model, in which the three parameters of two-wave LCS (correlation between individual scores at T1, cross-lagged paths between T1 scores and change score and change-to-change effect) were fixed to be zero, was first estimated. The parameters were gradually unfixed and changes in the model’s fit were observed to select the final model.

The baseline model presented a poor fit to the data (χ^2^_4_ = 29.08, *p* < 0.001; CFI = 0.268, SMRM = 0.170). The LCS scores for changes in difficulties in emotion regulation (μ_Δ_ = -4.33, *p* < 0.001) and for depressive symptoms (μ_Δ_ = -2.25, *p* < 0.001) were significant, suggesting a significant reduction in the levels of both variables from T1 to T2.

The model in which the correlation between T1 scores was free to vary showed greater improvement when compared to the baseline model (χ^2^_3_ = 12.57, *p* < 0.006, CFI = 0.72, SRMR = 0.08; Δχ^2^_1_ = 16.51, *p* < 0.001). Subsequently, a model in which the cross-lagged paths between T1 and change scores were free to vary was tested. Although the chi-square difference from the prior model was non-significant (Δχ^2^_1_ = 1.84, *p* < 0.05), the goodness-of-fit indices of the model improved slightly (χ^2^_2_ = 10.73, *p* < 0.004, CFI = 0.75, SRMR = 0.07). Finally, the model in which the change-to-change effects were free to vary was tested, showing a very good fit to the data (χ^2^_1_ = 2.09, *p* = 0.150, CFI = 0.97, SRMR = 0.055; Δχ^2^_1_ = 8.64, *p* < 0.01).

The parameter estimates of the final model (see Table 3) showed a positive association between changes in difficulties in emotion regulation and changes in depressive symptoms: a greater decrease in difficulties in emotion regulation levels was associated with a greater decrease in the levels of depressive symptoms. Women’s levels of difficulties in emotion regulation were positively associated with their levels of depressive symptoms at T1. Additionally, a negative and significant association between T1 scores on emotion regulation difficulties and change in depressive symptoms was found, suggesting that women’s higher levels of emotion regulation difficulties at T1 were associated with a greater decrease in depressive symptoms from T1 to T2.

#### Psychological Flexibility and Depressive Symptoms

A model examining the effect of changes in psychological inflexibility on changes in depressive symptoms over time was estimated.

The baseline model showed a very poor fit to the data (χ^2^_4_ = 37.75, *p* < 0.001, CFI = 0.42, SMRM = 0.238). The LCS scores showed a significant decrease over time of both psychological inflexibility (μ_Δ_ = -2.27, *p* = 0.002) and depressive symptoms (μ_Δ_ = -2.25, *p* < 0.001).

The unconstrained model in which T1 scores were allowed to correlate resulted in a significant improvement compared to the baseline model (χ^2^_3_ = 4.09, *p* = 0.25, CFI = 0.98, SMRM = 0.067; Δχ^2^_1_ = 33.66, *p* < 0.001). The model in which the cross-lagged paths between T1 and change scores were allowed to vary resulted in a non-significant chi-square difference from the prior model (Δχ^2^_1_ = 2.17, *p* > 0.05) but a slight improvement of goodness-of-fit indices (χ^2^_2_ = 1.92, *p* = 0.38, CFI = 1.00, SMRM = 0.029). Finally, the model in which the change-to-change effect was unconstrained resulted in a non-significant improvement in the chi-square statistic (Δχ^2^_1_ = 1.77, *p* > 0.05) and in a non-relevant improvement in the goodness-of-fit indices (χ^2^_1_ = 0.15, *p* = 0.699, CFI = 1.00, SMRM = 0.014).

This finding is congruent with the parameter estimates in the final model (see Table 3): no significant association was found between the women’s change in their levels of psychological inflexibility and the women’s change in their levels of depressive symptoms over time. Women’s levels of psychological inflexibility and depressive symptoms were associated at T1. No significant association was found between women’s psychological inflexibility at T1 and their levels of change in depressive symptoms.

#### Self-Compassion and Depressive Symptoms

A model examining the effect of changes in self-compassion on changes in depressive symptoms over time was estimated. The baseline model showed a very poor fit to the data (χ^2^_4_ = 31.06, *p* < 0.001, CFI = 0.16, SMRM = 0.193). The LCS scores showed a significant increase over time in the levels of self-compassion (μ_Δ_ = 3.43, *p* < 0.001) and a significant decrease over time in the levels of depressive symptoms (μ_Δ_ = -2.25, *p* < 0.001).

The model in which the correlation between T1 scores was unconstrained resulted in a great improvement when compared to the baseline model (Δχ^2^_1_ = 23.94, *p* < 0.001), although the goodness-of-fit indices continued to reveal a poor adjustment to data (χ^2^_3_ = 7.12, *p* = 0.006, CFI = 0.87, SRMR = 0.08). There was a slight improvement in the goodness-of-fit indices in the second model, in which the cross-lagged paths were unconstrained (χ^2^_2_ = 6.10, *p* = 0.004, CFI = 0.87, SRMR = 0.055), although the chi-square difference was non-significant (Δχ^2^_1_ = 1.10, *p* > 0.05). The final model, in which the change-to-change effects were unconstrained, showed the best fit to the data (χ^2^_1_ = 0.44, *p* = 0.510, CFI = 1.00, SRMR = 0.034; Δχ^2^_1_ = 5.66, *p* < 0.01).

The parameter estimates of the final model (see Table 3) showed a negative association between changes in self-compassion and changes in depressive symptoms: a greater increase in women’s self-compassion levels was associated with a greater decrease in women’s depressive symptoms. Women’s levels of self-compassion were associated with their depressive symptoms at T1 and women’s levels of self-compassion at T1 were not associated with the levels of change in their depressive symptoms over time.

## Discussion

Although this study was exploratory, its results represent an innovative contribution in the context of preventive interventions for PPD by providing some insight into the processes that underlie treatment response to the Be a Mom program. Be a Mom has shown preliminary evidence of efficacy in reducing depressive symptoms among at-risk women in the early post-partum period, and thus preventing the establishment of a clinical diagnosis of PPD (Fonseca et al., 2018a). Although Be a Mom was developed to target the enhancement of self-regulatory skills such as emotion regulation abilities, psychological flexibility and self-compassion (Fonseca et al., 2018c), further evidence was needed to establish whether these self-regulatory skills are effectively promoted within the program and whether they result in a significant reduction in the levels of depressive symptoms.

The first main finding of our study suggests that Be a Mom promotes the enhancement of emotion regulation abilities and self-compassion. Women who participated in Be a Mom showed a greater decrease in emotion regulation difficulties and a greater increase in self-compassion from baseline to post-intervention assessment when compared to women who did not participate in the program. In its first module, one of Be a Mom’s goals is to help women normalize and identify the diversity of their emotional experiences and promote its non-evaluative acceptance (Fonseca et al., 2018c). This may be of particularly importance, as transition to parenthood is usually viewed by society as a period of happiness and joy (Sutherland, 2010); the ability to accept that negative emotions are also part of the motherhood experience may help women to deal in a more adaptive way with the challenges posed by motherhood, as they are not focused in trying to avoid or control such negative emotions. The information and exercises proposed within the first module may have prompted women to increase the clarity (identification) and awareness of the emotions they experienced, to increase their acceptance of such emotions even if they were negative, and consequently to engage more in adaptive emotion regulation strategies and goal-directed behaviors, thereby enhancing their emotion regulation abilities. The enhancement of a more compassionate attitude toward themselves is promoted throughout the entire program, but particularly in the first and second modules of Be a Mom. A more self-compassionate attitude may help women to perceive the difficulties and challenges (e.g., difficulties in caregiving tasks, lack of time for themselves, changes in the relationship with the partner or friends) and negative experiences of motherhood (e.g., negative emotions and thoughts) as part of the human experience, and to act in a kind and warm way toward themselves when confronted with such difficult experiences, rather than being self-critical (Neff, 2009) and blaming themselves for not being “the perfect mother.” In the first module, women were educated about the negative effects of sociocultural myths of perfect motherhood and its associated unrealistic expectations (e.g., feelings of failure) and were offered some exercises that may have paved the way to a more self-compassionate approach to managing such expectations, accepting that they are vulnerable and human like all mothers. Similarly, in the second module, women were educated about the pervasive role of self-criticism in dealing with individual failures and suffering and were offered some exercises that aimed to promote a kinder and more compassionate attitude toward themselves in the maternal role, which may have contributed to increasing their levels of self-compassion.

Contrary to our expectations, there was a significant decrease in psychological inflexibility over time, but this decrease was equal in both groups, suggesting that Be a Mom did not contribute to the enhancement of psychological flexibility. Two reasons may help to explain these findings. On the one hand, psychological flexibility involves not only an accepting and non-judgmental way to deal with negative emotions and cognitions, which is promoted from the first module of Be a Mom but also the clear identification of an individual’s values and engagement in committed behaviors with such values (Hayes et al., 2006), which is targeted only in the third module. It is possible that the Be a Mom’s users who dropped out without completing the program may not have had the opportunity to learn such skills in an effective way. Additionally, the identification of parenthood values may be a time-consuming task, and the engagement in valued-based parenting behaviors is dependent upon clear identification. Therefore, it is possible that the strategies used to promote psychological flexibility within Be a Mom required more practice and time for their benefits to be observable. Further studies should examine whether the enhancement of psychological flexibility may be observable in follow-up assessments. On the other hand, it is possible that the questionnaire used to assess psychological inflexibility, which mainly targeted one of its dimensions (i.e., experiential avoidance), may not have adequately captured changes in the different dimensions of this construct (e.g., cognitive fusion, lack of value clarity), particularly considering the parenting context (Greene et al., 2015).

The results of this study provide valuable information on the relation between the core psychological processes targeted in the Be a Mom program and its observed outcomes. In particular, the second main finding of this study is that changes in difficulties in emotion regulation and in self-compassion over time were significantly associated with changes in depressive symptoms among women who participated in the Be a Mom program. First, baseline scores on self-regulatory skills and depressive symptoms were significantly associated, with women who presented poorer self-regulatory skills (more emotion regulation difficulties, high psychological inflexibility and low self-compassion) showing higher levels of depressive symptoms. These results were congruent with prior research that has suggested that poorer emotion regulation (Haga et al., 2012; Marques et al., 2018), higher psychological inflexibility (Zhu et al., 2015; Fonseca et al., 2018b) and poorer levels of self-compassion (Felder et al., 2016) may increase the likelihood of postpartum depressive symptoms. Without clinical intervention, these poorer self-regulatory skills may also contribute to the persistent nature of depressive symptoms over time, leading to the establishment of a clinical diagnosis of PPD. They, therefore, are important targets of preventive efforts.

Second, the enhancement in self-regulatory skills (emotion regulation abilities and self-compassion) in women who participated in Be a Mom seemed to exert a protective effect in the presence of PPD risk factors because it led to a reduction of depressive symptoms. The greater women’s ability to enhance such skills in the postpartum period, the greater their ability to deal with the private negative experiences (emotions and thoughts) associated with their parenting experience by being more aware and accepting of such emotions and by using more adaptive emotion regulation strategies (Haga et al., 2012). These women may also be able to adopt a kinder and more self-compassionate attitude toward their own experiences (Felder et al., 2016; Fonseca and Canavarro, 2018), which may help them to better address the unrealistic expectations of “perfect motherhood,” accept their vulnerable and human nature and be less judgmental toward themselves in the presence of motherhood-related negative experiences, both external and internal (e.g., thoughts/emotions) (Fonseca and Canavarro, 2018).

Finally, the results showed that the women’s scores on emotion regulation difficulties at baseline were associated with the degree of change in depression symptoms over time, suggesting that women who presented higher emotion regulation difficulties at baseline showed a greater decrease in depressive symptoms. One possible explanation for these results is that women who present more difficulties in regulating their emotions may be more prone to engage with the program’s information and exercises because they may find the program’s content more relevant to their needs, which may translate into significant benefits in terms of their depressive symptoms. However, further studies should examine this hypothesis.

Several limitations should be taken into consideration when interpreting our results. First, this study corresponds to a secondary analysis of the results of a pilot randomized trial conducted to evaluate Be a Mom’s feasibility and acceptability as well as to provide preliminary evidence of its efficacy (Fonseca et al., 2018a). However, randomization was not completely successful because the intervention and the control groups differed in terms of sociodemographic characteristics (marital status and income). The potential influence of such covariates was considered in preliminary analyses to minimize these limitations. Moreover, the dropout rate between baseline and post-intervention assessment may have influenced the results found because the women who dropped out of the study may have experienced fewer benefits in emotional adjustment than those who completed the study. However, following the intention-to-treat principles, we used a statistical approach that handled missing data to attempt to minimize the influence of study dropouts. Second, given the pilot nature of the study, the sample size and the number of assessment times were limited. Further studies with larger samples and additional follow-up assessments will allow further inspection of the processes of change over time in self-regulatory skills among women who participate in Be a Mom in both the short-term and the long-term.

Nonetheless, this study complements prior evidence of Be a Mom’s efficacy and shows that Be a Mom is effective not only in reducing depressive and anxiety symptoms (Fonseca et al., 2018a) but also in promoting targeted self-regulatory skills. Moreover, this study provides a valuable contribution to research on the processes of therapeutic change (Hayes et al., 2007; Kazdin, 2007) of (web-based) preventive CBT interventions for PPD by showing the link between changes in self-regulatory skills and changes in depressive symptoms among Be a Mom’s users.

## Author Contributions

AF designed the study, collaborated in the study implementation (randomization of participants), performed the data analyses, and wrote the manuscript. FM and SA were responsible for data collection and revised the final draft of the manuscript. RG and MC collaborated in the design of the study and in the revision of the final manuscript.

## Conflict of Interest Statement

The authors declare that the research was conducted in the absence of any commercial or financial relationships that could be construed as a potential conflict of interest.
